# Dynamic Immune–Nutritional Indices as Powerful Predictors of Pathological Complete Response in Patients with Breast Cancer Undergoing Neoadjuvant Chemotherapy

**DOI:** 10.3390/jcm15020418

**Published:** 2026-01-06

**Authors:** Emel Mutlu Ozkan, Ibrahim Karadag, Mevlude Inanc, Metin Ozkan

**Affiliations:** 1 Department of Medical Oncology Clinic, Faculty of Medicine, Erciyes University, 38280 Kayseri, Türkiye; mevludeinanc@hotmail.com (M.I.); metino@erciyes.edu.tr (M.O.); 2 Department of Medical Oncology Clinic, Ankara Atatürk Sanatorium Education and Research Hospital, 06290 Ankara, Türkiye; ikaradag58@gmail.com

**Keywords:** breast cancer, nutrition, inflammation, neoadjuvant chemotherapy, pathological complete response

## Abstract

**Background/Objectives**: Pathological complete response (pCR) is an established surrogate marker of neoadjuvant chemotherapy (NACT) efficacy in breast cancer; however, reliable predictors of pCR remain limited. Immune–inflammation- and nutrition-based biomarkers derived from routine blood tests may offer accessible tools for early assessments of treatment response. This study aimed to evaluate both baseline values and dynamic (Δ) changes in multiple immune–nutritional indices to determine their predictive performance with regard topCR. **Methods**: A retrospective analysis was conducted on 236 early breast cancer patients who received neoadjuvant chemotherapy. Pre-treatment (B), post-treatment (A), and Δ values were calculated for the prognostic nutritional index (PNI), advanced lung cancer inflammation index (ALI), hemoglobin–albumin–lymphocyte–platelet (HALP) score, systemic inflammation response index (SIRI), pan-immune–inflammation value (PIIV), global immune–nutrition-information index (GINI), nutritional risk index (NRI), and related biomarkers. Associations with pCR were examined using chi-square testing and univariate logistic regression, and diagnostic performance was assessed through receiver operating characteristic (ROC) analysis. **Results**: pCR was achieved in 116 patients (49.2%). Logistic regression identified the NRI (OR = 2.336), ΔGINI (OR = 2.323), ALI (OR = 1.318), PNI (OR = 1.365), HALP score (OR = 1.217), ΔSIRI (OR = 2.207), and ΔPIIV (OR = 2.001) as significant predictors. ROC analysis showed that the NRI (AUC = 0.840) and ΔGINI (AUC = 0.807) were the strongest discriminators of pCR. In aLASSO (Least Absolute Shrinkage and Selection Operator)-penalized logistic regression with 10-fold cross-validation, the NRI and ΔGINI emerged as independent predictors of pCR (OR = 1.28 and OR = 1.23, respectively), showing acceptable calibration particularly in the moderate-to-high probability range. **Conclusions**: Both baseline and **Δ** immune–nutritional biomarkers predict pCR following NACT in breast cancer. The NRI and ΔGINI demonstrated the best diagnostic performance, whereas ΔSIRI and ΔPIIV also showed meaningful associations. Easily obtainable, low-cost indices—particularly **Δ** markers—may support the early identification of responders and facilitate more personalized therapeutic decision-making in breast cancer management.

## 1. Introduction

Breast cancer ranks among the most frequently diagnosed cancers in women globally and remains the primary cause of cancer-related mortality in women. It represents 30% of all new cancer cases and 15% of cancer deaths in women, based on current data [1]. International guidelines recommend treatments such as surgery, chemotherapy, hormone therapy, targeted therapy, immunotherapy, and radiotherapy, with nutritional status also influencing prognosis and outcomes. Surgery is often unsuitable for breast cancer patients with axillary lymph node metastasis. Neoadjuvant chemotherapy (NACT) before local therapy is increasingly used, improving breast conservation rates and reducing the risk of distant metastasis [2]. Not all breast cancer patients achieve complete remission after NACT. Pathological complete response (pCR) is the most reliable predictor of NACT efficacy and is linked to improved overall survival, particularly in HER2-positive and triple-negative subtypes, though its benefit in luminal A tumors is less clear [3]. Nevertheless, reliable indicators to accurately assess tumor response and treatment effectiveness prior to personalized therapy are still lacking.

Loss of appetite, weight loss, and cachexia are common in cancer patients. Affecting 50–80% of cases and linked to 20–40% of cancer deaths, cachexia worsens the prognosis by accelerating disease progression and reducing treatment efficacy [4,5]. Therefore, identifying more practical and accessible indicators to assess the impact of nutritional status on treatment outcomes and disease prognosis in breast cancer is critically important.

Recent studies highlight the impact of the tumor microenvironment and nutrition on cancer prognosis. Shaped by inflammatory cells and mediators, the microenvironment influences tumor initiation, progression, and treatment response. Peripheral blood cells such as neutrophils, lymphocytes, monocytes, and platelets can provide insights into intratumoral immunity through derived laboratory parameters [6]. The advanced lung cancer inflammation index (ALI), neutrophil-to-lymphocyte ratio (NLR), platelet-to-lymphocyte ratio (PLR), pan-immune–inflammation value (PIIV), prognostic nutritional index (PNI), systemic immune–inflammation index (SII), hemoglobin–albumin–lymphocyte and platelet (HALP) score, systemic inflammation response index (SIRI), nutritional risk index (NRI), and global immune–nutrition-information index (GINI) have been used previously to determine survival in many cancer types. The effectiveness of these pre-chemotherapy scores in predicting survival has been evaluated in previous studies. In addition, a focused literature scan confirmed that prior studies have evaluated only static forms of the SIRI, PIIV, and GINI, whereas their dynamic (Δ) derivatives during neoadjuvant chemotherapy in breast cancer have not been systematically investigated to date, further supporting the translational relevance of our findings [5,7,8,9,10,11,12].

These Δ indices integrate dynamic immune reprogramming rather than static inflammation. Neutrophils and monocytes promote tumor-supportive inflammation and immunosuppression, whereas lymphocytes—particularly CD8^+^ T cells—mediate antitumor immunity. ΔGINI, ΔSIRI, and ΔPIIV simultaneously integrate systemic inflammation, nutritional status, and immune suppression. By incorporating CRP, neutrophils, monocytes, platelets, albumin, and lymphocytes, ΔGINI reflects the interconnected inflammation–nutrition–immunity axis that fundamentally drives tumor progression, systemic metabolic dysfunction, and treatment responsiveness. CRP serves as a key acute-phase reactant signaling cytokine-mediated inflammation, while neutrophils and monocytes contribute to tumor-promoting myeloid activity through the release of proteolytic enzymes, reactive oxygen species, and immunosuppressive mediators. Platelets not only regulate coagulation but also shield circulating tumor cells, facilitate metastatic spread, and amplify inflammatory cascades. Conversely, lymphocytes—particularly cytotoxic T cells—represent the core effector arm of antitumor immune surveillance, and reductions in lymphocyte counts indicate impaired adaptive immunity. Serum albumin captures the nutritional and inflammatory burden, reflecting hepatic synthetic capacity and systemic catabolism [13,14,15,16,17,18,19,20]. Consequently, favorable treatment-induced shifts in ΔSIRI, ΔPIIV, and ΔGINI likely represent the attenuation of tumor-supportive inflammation and restoration of effective antitumor immune surveillance, providing a biologically plausible explanation for their association with enhanced chemosensitivity and the achievement of pCR.

We hypothesized that ΔPNI, ΔSII, ΔALI, ΔSIRI, ΔHALP, ΔPIIV, ΔNLR, ΔPLR, ΔGINI, and the NRI, which represent changes in nutritional and immune status before and after NACT, may more accurately reflect patients’ responses to chemotherapeutic agents. These scores reflect the combined changes in inflammation and nutritional status occurring before (B) and after (A) NACT. In this study, we are the first to evaluate the ΔALI, ΔSIRI, ΔGINI, and ΔPIIV scores as novel markers to identify the most effective parameters for predicting pCR in breast cancer patients undergoing NACT.

## 2. Materials and Methods

### 2.1. Study Design and Patient Characteristics

A total of 236 patients diagnosed with breast cancer who received NACT at Erciyes University Faculty of Medicine, Turkey, between January 2016 and March 2023 were included in this study. The faculty’s pathology department assessed the patients’ pathological response rates. Clinicopathological data, complete blood counts, and biochemical test results were retrieved from medical records. All blood samples were collected within one week prior to neoadjuvant therapy. Post-NACT blood samples were collected in a standardized manner within 28–35 days after completion of the final chemotherapy cycle and before surgery.

Patients with active infections at baseline or after NACT, those requiring chronic systemic steroid therapy, and those who received granulocyte colony-stimulating factor (G-CSF) after NACT were excluded from the study. No patients received formal nutritional intervention, either enteral or parenteral, during the NACT period. All patients had an Eastern Cooperative Oncology Group (ECOG) performance status of 0–1 at treatment initiation. Moreover, patients with major uncontrolled comorbidities, including advanced cardiac failure, renal failure, or autoimmune inflammatory diseases, were not included in the cohort.

The following indices were determined using the specified formulas:

**NLR:** Absolute neutrophil count (10^3^/mm^3^)/Absolute lymphocyte count (10^3^/mm^3^)

**BMI (body mass index):** Weight/height^2^ (kg/m^2^)

**ALI:** Serum albumin (g/L)/10 × BMI/NLR

**SII:** NLR × Platelet count (10^3^/mm^3^)

**PNI:** [Serum albumin (g/L)] + [Absolute lymphocyte count (10^3^/mm^3^) × 5]

**PLR:** Platelet count (10^3^/mm^3^)/Absolute lymphocyte count (10^3^/mm^3^)

**SIRI:** NLR × Absolute monocyte count (10^3^/mm^3^)

**PIIV:** SIRI × Platelet count (10^3^/mm^3^)

**HALP**: [Hemoglobin (g/dL) × albumin (g/L) × Absolute lymphocyte count (10^3^/mm^3^)]/Absolute platelet count (10^3^/mm^3^).

**Ideal body weight:** Height − 100 − [(Height − 150)/2.5]

**NRI:** [1.519 × serum albumin level (g/L)] + [41.7 × (present/ideal body weight)]

**GINI:** [C-reactive protein× Platelet count (10^3^/mm^3^) × Absolute monocyte count (10^3^/mm^3^) × Absolute neutrophil count (10^3^/mm^3^)]/[Serum albumin (g/L) × Absolute lymphocyte count (10^3^/mm^3^)].

**Δ:** (A) NACT score − (B) NACT score

Relative percentage changes were not used in the analysis.

### 2.2. Statistics

Statistical analyses were performed using SPSS 23.0 software. The associations between clinicopathological and laboratory parameters were assessed with the chi-square, Mann–Whitney U, and Fisher’s exact tests. Logistic regression analysis was applied to identify factors influencing the therapeutic response in breast cancer patients. The prognostic impact of inflammatory markers associated with pCR was evaluated using univariate logistic regression analysis. A *p*-value of <0.05 was considered to indicate statistical significance. Variables with a *p*-value of <0.05 were included in an ROC analysis to determine predictive factors for pCR. Due to multicollinearity among the inflammatory biomarkers predicting pCR, LASSO (Least Absolute Shrinkage and Selection Operator) penalized logistic regression analysis was applied instead of conventional multivariable modeling. A combined ROC analysis was performed for the variables that remained significant in the LASSO penalized logistic regression analysis.

## 3. Results

**Figure 1:**
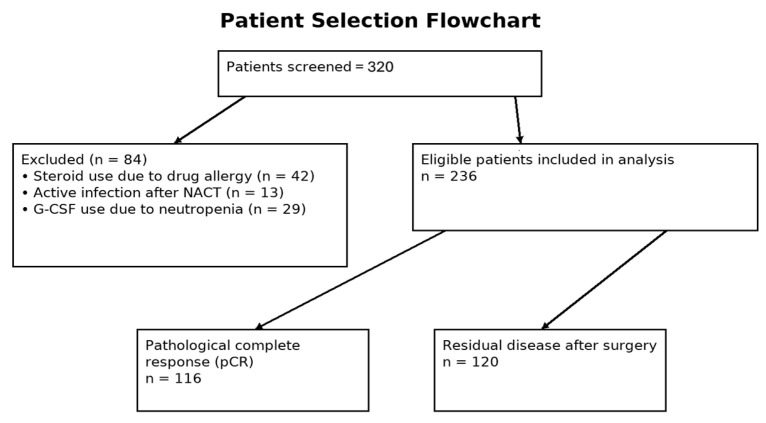
Patient selection flowchart.

A total of 236 female patients with breast cancer were included in this study. The patient selection flowchart is shown in Figure 1.

The median age of the patients was 49 years, and 120 (50.8%) of them were postmenopausal. Following NACT, pCR was achieved in 116 patients (49.2%) (Table 1).

All patients received four cycles of anthracycline plus cyclophosphamide followed by twelve cycles of paclitaxel as their NACT. For HER2-positive patients, trastuzumab was added to the paclitaxel regimen. Carboplatin was added to paclitaxel for only 10 (4.2%) triple-negative patients. Unfortunately, pembrolizumab and pertuzumab, which are now considered standard treatments, could not be administered at that time because they were not covered by the national health insurance system.

The inflammatory scores (B) NACT and (A) NACT and their Δ values were analyzed according to the presence of pCR. There was a statistically significant difference in (B) PNI values according to the presence of pCR (*p* = 0.041). The median (B) PNI was 57 in patients with pCR and 46.25 in those with residual disease. Similarly, (B) HALP values differed significantly between the two groups (*p* = 0.015), with medians of 49.91 in patients with pCR and 42.2 in those with residual disease. (B) ALI values were also significantly higher in patients with pCR (median 78.18 vs. 59.94, *p* < 0.001). No significant associations were found between pCR and other (B) inflammatory scores (*p* > 0.05). (A) GINI values were significantly lower, while NRI and (A) ALI values were significantly higher in patients with pCR (*p* = 0.042, *p* < 0.001, and *p* = 0.001, respectively). The median (A) GINI value was 160.01 in the pCR group and 203.79 in the residual group, while the (A) ALI values were 62.36 and 31.81, respectively. Regarding Δ values, significant differences were observed in ΔSIRI (*p* = 0.029), ΔPIIV (*p* = 0.029), and ΔGINI (*p* = 0.009). The median ΔSIRI, PIIV, and GINI values were 0.13, 21.2, and 16.89 in the pCR group, compared with −0.1, −39.92, and −32.51 in the residual group. No other Δ inflammatory indices were significantly associated with pCR (*p* > 0.05) (Table 2).

The prognostic impact of inflammatory markers associated with pCR was evaluated using univariate logistic regression analysis. Due to multicollinearity among the variables, multivariate analysis was not performed. Therefore, instead of applying a conventional multivariable logistic regression model, variables that showed statistically significant associations in univariate logistic regression analyses were further evaluated using LASSO penalized logistic regression, which effectively addresses multicollinearity among correlated predictors. The univariate analysis showed that higher NRI (OR = 2.336, *p* < 0.001), (B) PNI (OR = 1.365, *p* = 0.023), HALP (OR = 1.217, *p* = 0.025), and ALI (OR = 1.318, *p* < 0.001) values were significantly associated with an increased likelihood of achieving pCR. Similarly, higher ΔSIRI (OR = 2.207, *p* = 0.001), ΔPIIV (OR = 2.001, *p* = 0.020), and ΔGINI (OR = 2.323, *p* = 0.001) values were associated with an increased likelihood of achieving pCR (Table 3).

**Figure 2:**
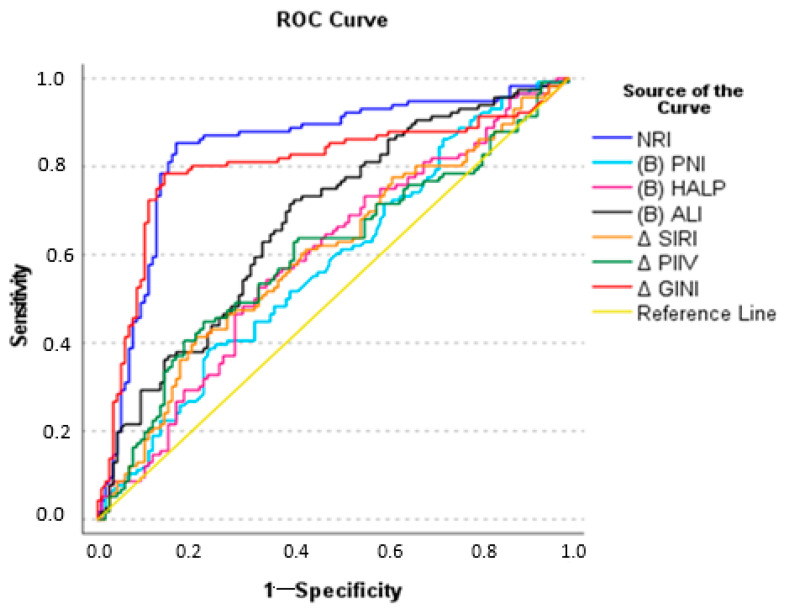
ROC curves of immune nutritional scores from univariate logistic regression analysis.

The diagnostic performance of parameters associated with pCR was evaluated by means of ROC analysis, and the values were found to have statistically significant diagnostic accuracy (*p* < 0.05). The NRI had a cutoff of ≥126.1, with an area under the curve (AUC) of 0.840 (95% CI: 0.784–0.895), sensitivity of 85.3%, and specificity of 83.3%. ΔGINI had a cutoff of ≥2.21 with an AUC of 0.807 (95% CI: 0.609–0.945), sensitivity of 80.4%, and specificity of 80.1% (Table 4). The ROC analysis indicated that the NRI and ΔGINI were good discriminators for pCR, while other variables showed low discriminatory ability (Figure 2).

The LASSO model was constructed using 10-fold cross-validation, and the model’s performance was assessed based on both the penalty parameter that minimized the cross-validated error (λ_min = 0.029) and the more parsimonious and stable penalty parameter selected according to the one-standard-error (1-SE) rule (λ_1se = 0.119).

Under the parsimonious λ_1se model, the number of predictors retained in the model was substantially reduced, and only the NRI and ΔGINI remained with non-zero coefficients, while all other immune–nutritional biomarkers were shrunk to zero and excluded from the model. This finding suggests that the NRI and ΔGINI may be independently associated with pCR, beyond the effects of other correlated inflammatory and nutritional indices. In the λ_1se model, the estimated coefficient for the NRI was β = 0.257, corresponding to an OR of 1.28, whereas the coefficient for ΔGINI was β = 0.218, corresponding to an OR of 1.23. These results indicate that each one-unit change in ΔGINI is associated with an approximately 23% increase in the likelihood of achieving pCR, while each one-unit change in the NRI is associated with an approximately 28% increase in the likelihood of pCR.

**Figure 3:**
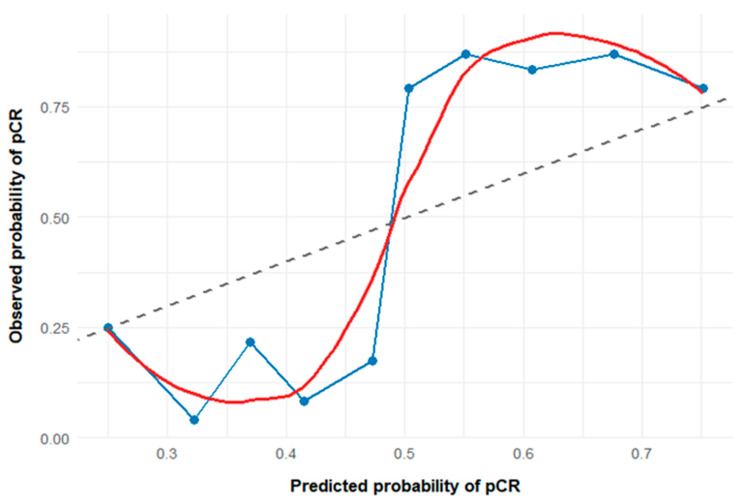
The calibration plot of the LASSO logistic regression model. Points represent observed versus predicted pCR probabilities across deciles of predicted risk. The dashed line indicates perfect calibration, while the solid curve represents LOESS-smoothed calibration.

The model intercept was not interpreted due to its limited clinical relevance. The predictive performance and calibration of the model were assessed using calibration analysis. As shown in Figure 3, the calibration plot demonstrated good agreement between predicted probabilities and observed pCR rates, particularly within the clinically relevant moderate-to-high probability range. Minor miscalibration was observed at lower predicted probabilities, which was expected given the decile-based grouping and sample size limitations. Overall, the model exhibited acceptable calibration for clinically meaningful pCR risk estimation. Using the variables selected in the LASSO penalized logistic regression (NRI and ΔGINI), a combined ROC analysis was performed to predict pCR. The model incorporating both variables demonstrated high discriminative performance. The AUC, calculated using 10-fold cross-validation, was 0.842 (bootstrap 95% CI: 0.789–0.895). At the optimal cutoff value determined by the Youden index, the model achieved a sensitivity of 87.1% and a specificity of 80.0%.

## 4. Discussion

In this study, we conducted a comprehensive evaluation of immune–inflammatory- and nutrition-based biomarkers both at baseline and through their dynamic changes during NACT to determine their predictive value for pCR in breast cancer. Our findings demonstrated that several pre-treatment indices, including (B) PNI, (B) HALP, and (B) ALI, as well as the NRI and post-treatment levels of (A) ALI and (A) GINI, were significantly associated with pCRin a univariate logistic regression analysis. These results are consistent with those of prior studies showing that nutritional and immune–inflammatory biomarkers influence treatment response in breast cancer and other malignancies [5,6,21,22,23]. More importantly, Δ parameters (ΔSIRI, ΔPIIV, and ΔGINI) emerged as novel and sensitive indicators of treatment response. Among all indices, the NRI and ΔGINI showed the highest discriminatory accuracy in ROC analysis, underscoring their potential utility as practical and non-invasive predictors of chemotherapy effectiveness.

Our results align with earlier evidence demonstrating the prognostic and predictive significance of host nutritional and immune–inflammatory status in various cancers. Pre-treatment PNI, HALP, and ALI values have been repeatedly shown to correlate with survival and pCR outcomes in breast cancer and other solid tumors. Prior studies emphasized that higher PNI values reflect better lymphocyte-mediated antitumor immunity and preserved albumin levels, both of which facilitate improved response to systemic therapy. Consistent with our findings, the ALI has been validated as a prognostic marker in lung and gastrointestinal cancers and recently gained attention in breast cancer populations. Similarly, the HALP score is increasingly recognized as a prognostic indicator due to its incorporation of hemoglobin (oxygen transport), albumin (nutritional status), lymphocytes (immune response), and platelets (inflammatory activity) [6,7,21,23,24]. There are several biologically plausible explanations for the observed associations. Systemic inflammation and immune dysregulation impair chemotherapy response by modifying the tumor microenvironment, increasing cytokine-mediated resistance, and suppressing cytotoxic lymphocyte activity. Neutrophils promote tumor progression by releasing various inflammatory mediators, while platelets can shield cancer cells from natural killer cell-mediated destruction. In contrast, lymphocytes support antitumor immunity and play a central role in immune surveillance. Tumor-infiltrating lymphocytes, mainly CD4^+^ and CD8^+^ T cells, suppress tumor growth by inducing cancer cell apoptosis. Therefore, higher lymphocyte levels strengthen antitumor immune responses within the tumor microenvironment and help inhibit cancer progression. Nutritional deterioration, often exacerbated during chemotherapy, leads to hypoalbuminemia, sarcopenia, and metabolic imbalance, all of which weaken immune function and decrease tolerance to chemotherapeutic agents [25,26]. Changes in Δ inflammatory indices likely reflect the degree to which a patient’s immune system recovers, rebalances, or adapts in response to cytotoxic therapy. Chemotherapy induces both immunogenic cell death and transient immunosuppression, and the net biological effect on systemic immunity can be captured by Δ changes rather than static measurements. Improvements in ΔSIRI or ΔGINI may indicate the attenuation of neutrophil-driven pro-inflammatory signaling, a reduction in monocyte-mediated macrophage polarization toward tumor-supportive phenotypes, and partial restoration of lymphocyte-mediated antitumor activity. Such shifts suggest a transition from a protumoral, myeloid-dominant immune environment toward a more balanced and responsive systemic immunologic state. Composite indices like the GINI and PIIV are particularly informative because they integrate multiple biological pathways simultaneously—acute-phase inflammation (CRP), myeloid cell activity (neutrophils and monocytes), thrombocyte-driven immune evasion (platelets), adaptive immunity (lymphocytes), and nutritional/metabolic status (albumin). This multidimensional integration may allow these Δ markers to detect subtle physiological changes induced by chemotherapy that would not be evident in isolated hematologic parameters. The Δ immune–nutritional indices therefore capture treatment-induced immune recalibration, rather than reflecting static host characteristics. Specifically, ΔSIRI quantifies shifts in the neutrophil–monocyte–lymphocyte axis, ΔPIIV integrates platelet-mediated tumor shielding with systemic inflammation, and ΔGINI reflects the combined burden of inflammation, immune suppression, and nutritional deterioration. Similarly, ΔALI reflects dynamic interactions between systemic inflammation, body composition, and nutritional status, all of which modulate chemotherapy tolerance and tumor microenvironment responsiveness. Together, these Δ indices offer a biologically coherent framework for assessing the host’s evolving immune–nutritional state during neoadjuvant treatment. Together, these Δ markers may serve as sensitive indicators of how effectively chemotherapy modulates the tumor–host interaction, thereby providing a biologically coherent explanation for their association with enhanced chemosensitivity and the achievement of pathological complete response. Studies in the literature have consistently demonstrated that reductions in inflammatory signaling, restoration of lymphocyte function, and improvements in metabolic balance enhance chemosensitivity and increase the likelihood of achieving pathological complete response, supporting the relevance of these Δ biomarkers as novel predictors of treatment efficacy in breast cancer [27,28,29,30,31]. These mechanisms underscore that the interaction between systemic physiology and tumor biology is dynamic, not static. Treatment response in oncology is shaped not only by tumor biology but also by a broad range of multidimensional patient-related factors. In many healthcare settings, the effectiveness of clinical interventions is influenced by human-centered elements such as workforce competencies, process quality, communication, and organizational performance. Evidence further indicates that, as in other sectors, strategic planning efforts play a substantial role in determining healthcare performance [32]. This perspective suggests that **Δ** immuno-nutritional responses are driven not only by biological mechanisms but also by system-level and human-dependent aspects of care. Additionally, improvements in healthcare quality may positively support treatment success through their effects on nutritional status and immuno-nutritional indicators. Monitoring Δ values may therefore allow clinicians to identify early on—possibly even during NACT—whether a patient is biologically progressing toward a complete pathological response.

The pCR rate in our study (49.2%) is consistent with reported outcomes for anthracycline–taxane regimens, which typically range from 30% to 60% [33]. It is important to acknowledge that the absence of pertuzumab and pembrolizumab—now standard for HER2-positive and triple-negative breast cancer—may have influenced the absolute pCR rates, but this does not diminish the internal validity of our biomarker analysis. Instead, it offers a valuable baseline for comparison with future cohorts receiving modern immunotherapy-based NACT.

Our observation that the NRI is the strongest predictor of pCR (AUC in univariate logistic regression analysis: 0.840) is consistent with the findings of Chen et al., who reported the NRI as an independent survival predictor in breast cancer patients undergoing NACT [5]. Studies in the literature have demonstrated that systemic nutritional and metabolic status plays a fundamental role in modulating antitumor immunity, inflammatory signaling, and treatment responsiveness. Evidence from pre-clinical studies on calorie restriction (CR) further supports this concept, showing that reduced caloric intake—without inducing malnutrition—can enhance the efficacy of chemotherapy, radiotherapy, and immunotherapy by improving insulin sensitivity, promoting autophagy, lowering circulating glucose levels, and suppressing pro-inflammatory and angiogenic pathways. These CR-related mechanisms align closely with the biological domains captured by immune–nutritional indices such as the NRI and ΔGINI, which similarly reflect inflammation, metabolic balance, and immune function. Thus, our findings are consistent with emerging research indicating that host nutritional modulation may directly influence chemosensitivity and therapeutic outcomes in breast cancer [34,35,36]. These parallels reinforce the notion that nutritional and systemic inflammatory burdens play a central role in determining chemotherapeutic responsiveness.

The novelty of our study stems from the evaluation of Δ indices, which capture physiological shifts occurring throughout NACT. While several previous studies examined static inflammatory markers, very limited research has assessed whether treatment-induced changes in systemic inflammation and nutritional status influence treatment response [6,37]. Our findings showed that favorable variations—particularly rises in ΔSIRI, ΔPIIV, and ΔGINI—were significantly associated with achieving pCR. This suggests that the biological adaptations to chemotherapy, rather than baseline status alone, may more directly reflect tumor chemosensitivity. Additionally, the NRI and ΔGINI emerged as independent predictors in the penalized multivariable analysis. In the ROC analysis in which the NRI and ΔGINI were evaluated in combination, the AUC was 0.842, with a sensitivity of 87.1% and a specificity of 80.0%. These results demonstrate that the combined evaluation of the NRI and ΔGINI provides superior discriminative performance for predicting pCR compared with the use of individual biomarkers alone. The LASSO approach effectively addressed multicollinearity and enabled the development of a more parsimonious and clinically interpretable model.

To our knowledge, this is the first study to investigate ΔALI, ΔPIIV, and ΔGINI in breast cancer patients treated with NACT, providing an important contribution to the evolving landscape of host-related biomarkers. All evaluated biomarkers are inexpensive to determine, easily measurable through standard laboratory tests, and widely available in clinical oncology settings. These **Δ** indices could help identify non-responders earlier, potentially enabling timely modifications such as switching regimens, incorporating immunotherapy, or intensifying supportive care. These parameters show that modifiable, targeted nutritional support during NACT may enhance treatment response—an area deserving further prospective evaluation. These biomarkers could complement radiological assessments and molecular subtype classification, contributing to a more holistic and individualized prediction model for pCR.

### 4.1. Strengths and Novel Contributions

This study has several strengths:

- It used a relatively large sample size (n = 236) with homogeneous treatment regimens.
- It included simultaneous assessments of multiple immune–nutritional indices, allowing direct comparison of their predictive performance.
- It introduces dynamic Δ metrics—particularly ΔSIRI, ΔPIIV, and ΔGINI—as novel predictors for pCR.
- It provides the first evidence supporting ΔGINI as an effective discriminative marker with an AUC above 0.80.
- Following LASSO penalized logistic regression analysis for predicting pCR, a combined ROC analysis was performed. The model incorporating both the NRI and ΔGINI demonstrated high discriminative performance (AUC0.842, sensitivity 87.1%, and specificity 80.0%).

These contributions highlight the potential value of systemic inflammation-based composite scores in improving predictive modeling of NACT response.

### 4.2. Limitations

This study has several important limitations. Its retrospective single-center design introduced potential selection bias and limits its generalizability. The absence of long-term survival data precluded assessment of whether **Δ** immune–nutritional indices also predict disease-free or overall survival. In addition, the lack of an external validation cohort and reliance on internal validation restrict the robustness and generalizability of the findings. The absence of contemporary immunotherapy and dual HER2 blockade further limits extrapolation to current treatment settings. Finally, unmeasured factors such as subclinical infection, occult inflammation, or unrecorded nutritional fluctuations may have influenced biomarker levels. The wide confidence intervals in the ROC analysis, particularly for ΔGINI, likely reflect biological heterogeneity and sample size constraints, underscoring the need for prospective multicenter validation with adequately powered penalized models.

## 5. Conclusions

In conclusion, our strengthened analysis indicates that both baseline and **Δ** immune–nutritional markers are valuable predictors of pCR in breast cancer patients undergoing NACT. The NRI and ΔGINI demonstrated the highest discriminative power, while ΔSIRI and ΔPIIV also showed meaningful associations. These biomarkers offer a promising avenue for early, low-cost, and non-invasive prediction of chemotherapy response and may contribute to personalized treatment approaches in modern breast cancer care.

## Figures and Tables

**Table 1:** Clinicopathological characteristics of patients.

Variable	n/Mean ± SD	%/Median (Min.–Max.)
**Age**	50 ± 10.66	49 (27–76)
**BMI**	30.4 ± 5.82	29.76 (18.36–49.12)
**Menopausal Status**		
Premenopausal	120	50.8
Postmenopausal	116	49.2
**Ideal Body Weight**	55.14 ± 3.65	55.4 (44.6–63.2)
**NRI**	126.29 ± 11.83	126.1 (92.22–162.96)
**Clinical T Stage**		
T1	26	11
T2	152	64.4
T3	33	14
T4	25	10.6
**Clinical Lymph Node**		
N0	35	14.8
N1	146	61.9
N2	47	19.9
N3	8	3.4
**Molecular Subtype**		
Luminal A	27	11.4
Luminal B	92	39
HER2+	81	34.3
Triple-	36	15.3
**Ki-67 Index**		
<20%	55	23.3
>20%	181	76.7
**Grade**		
Grade 1	5	2.1
Grade 2	164	69.5
Grade 3	67	28.4
**Operation Type**		
BCS	86	36.4
MRM	150	63.6
**Pathological Response**		
pCR	116	49.2
Residual mass	120	50.8

**BMI:** body mass index; **NRI:** nutritional risk index; **BCS:** breast-conserving surgery; **MRM:** modified radical mastectomy; **pCR:** pathological complete response.

**Table 2:** Analysis of patient characteristics according to pCR status.

Variable	pCR (*n* = 116) Median (Min–Max)/%	Residual Mass (*n* = 120) Median (Min–Max)/%	Test st.	*p* ^m^
**(B) PNI**	57 (41.8–69.05)	46.25 (43.8–69.6)	−2.041	**0.041 ^m^**
**(B) SII**	596.93 (150.11–2164.45)	535.15 (167.29–3733.52)	−1.540	0.124 ^m^
**(B) HALP**	49.91 (10.46–120.39)	42.2 (13.09–104.88)	−2.428	**0.015 ^m^**
**(B) SIRI**	1.06 (0.24–6.62)	0.96 (0.14–5.85)	−1.102	0.270 ^m^
**(B) PIIV**	303.01 (63.05–1303.54)	274.37 (37.61–1568.08)	−0.993	0.321 ^m^
**(B) NLR**	2.04 (0.68–11.83)	1.98 (0.83–13.93)	−1.301	0.193 ^m^
**(B) PLR**	140 (60–630)	130 (60–460)	−1.470	0.142 ^m^
**(B) GINI**	217.19 (13.52–16693.12)	204.56 (5.85–1627.7)	−0.038	0.970 ^m^
**(B) ALI**	78.18 (8.45–205.78)	59.94 (13.15–212.88)	−4.712	**<0.001 ^m^**
**(A) PNI**	52.58 (36.6–431.65)	53.75 (40.6–69.45)	−0.523	0.601 ^m^
**(A) SII**	626.34 (91.14–2753.51)	569.18 (91.14–1733.31)	−0.260	0.795 ^m^
**(A) HALP**	31.88 (7.05–191.35)	35.11 (12.36–107.54)	−1.311	0.190 ^m^
**(A) SIRI**	1.01 (0.15–7.46)	1.1 (0.33–6.15)	−1.281	0.200 ^m^
**(A) PIIV**	262.41 (27.34–2372.04)	308.07 (27.34–2208.47)	−1.220	0.223 ^m^
**(A) NLR**	2.23 (0.68–8.55)	2.14 (0.89–8.6)	−0.178	0.858 ^m^
**(A) PLR**	0.17 (0.05–0.59)	0.16 (0.05–0.39)	−1.337	0.181 ^m^
**(A) GINI**	160.01 (7.3–3202.77)	203.79 (12.01–2936.64)	−2.029	**0.042 ^m^**
**(A) ALI**	62.36 (53.95–14.8)	31.81 (68.74–18.76)	−3.299	**0.001 ^m^**
**ΔPNI**	−4.1 (−15–8.5)	−2.43 (−18.45–375.3)	−1.541	0.123 ^m^
**ΔSII**	49.95 (−3194.91–1348.85)	−28.11 (−1806.87–2244.15)	−1.647	0.100 ^m^
**ΔHALP**	−13.4 (−85.7–40.71)	−10.35 (−93.5–152.17)	−1.704	0.088 ^m^
**ΔSIRI**	0.13 (−4.73–3.41)	−0.1 (−5.47–6.78)	−2.605	**0.029 ^m^**
**ΔPIIV**	21.2 (−1309.55–1355.3)	−39.92 (−979.67–2169.02)	−2.623	**0.029 ^m^**
**ΔNLR**	0.17 (−11.59–6.92)	0.07 (−10.06–6.66)	−1.326	0.185 ^m^
**ΔPLR**	0.02 (−0.32–0.29)	0.02 (−0.51–0.48)	−0.060	0.952 ^m^
**ΔGINI**	16.89 (−1200.52–2335.76)	−32.51 (−16,658.94–2245.17)	−2.064	**0.009 ^m^**
**ΔALI**	−10.72 (−130.97–80.01)	−3.98 (−138.9–592.58)	−1.844	0.065 ^m^
**NRI**	132.82 (92.22–162.96)	119.08 (96.58–153.43)	−9.014	**<0.001 ^m^**
**Age**	52 (27–75)	45.5 (27–76)	−3.761	**<0.001 ^m^**
**BMI**	34.08 (21.94–49.12)	26.94 (18.36–45.37)	−8.421	**<0.001 ^m^**
**Menopausal Status**				
Premenopausal	44 (37.9)	76 (63.3)	15.23	**<0.001 ^x^**
Postmenopausal	72 (62.1)	44 (36.7)		
**IHC**				
Luminal A	12 (10.3)	15 (12.5)	2.861	0.581 ^x^
Luminal B	44 (37.9)	48 (40)		
HER2+	43 (37.1)	38 (31.7)		
Triple-	17 (14.7)	19 (15.8)		
**Ki-67 Index**				
≤20%	24 (20.7)	31 (25.8)	0.873	0.350 ^x^
>20%	92 (79.3)	89 (74.2)		
**Grade**				
Grade 1	3 (2.6)	2 (1.7)	5.973	**0.042 ^f^**
Grade 2	72 (62.1)	92 (76.7)		
Grade 3	41 (35.3)	26 (21.7)		

**m:** Mann–Whitney U test; **min:** minimum; **max:** maximum; **Test st.**: test statistic; **x:** Pearson chi-square test; **f:** Fisher’s exact test; **pCR:** pathological complete response; **Δ:** (A) − (B); **(A):** after neoadjuvant chemotherapy; **(B):** before neoadjuvant chemotherapy; **PNI:** prognostic nutritional index; **SII:** systemic immune–inflammation index; **HALP:** hemoglobin–albumin–lymphocyte and platelet; **SIRI:** systemic inflammation response index; **PIIV:** pan-immune–inflammation value; **NLR:** neutrophil-to-lymphocyte ratio; **PLR:** platelet-to-lymphocyte ratio; **GINI:** global immune–nutrition-information index; **ALI:** advanced lung cancer inflammation index; **NRI:** nutritional risk index; **BMI:** body mass index. Bold *p* values are statistically significant.

**Table 3:** Univariate logistic regression analysis of inflammatory scores associated with pCR.

Variable	SE	Wald	OR (%95 CI)	*p*
**NRI**	0.018	51.816	2.336 (1.097–4.176)	**<0.001**
**(B) PNI**	0.028	5.149	1.365 (1.009–1.125)	**0.023**
**(B) HALP**	0.007	5.052	1.217 (1.002–1.031)	**0.025**
**(B) ALI**	0.004	16.883	1.318 (1.009–1.027)	**<0.001**
**(A) ALI**	0.003	1.183	1.004 (0.997–1.01)	0.277
**(A) GINI**	0	1.202	1 (1–1.001)	0.273
**ΔSIRI**	0.134	1.980	2.207 (1.929–4.568)	**0.001**
**ΔPIIV**	0.012	1.785	2.001 (1.123–4.001)	**0.020**
**ΔGINI**	0.006	1.670	2.323 (1.006–3.123)	**0.001**

**SE:** standard error; **OR:** odds ratio; **CI:** confidence interval; **pCR:** pathological complete response; **NRI:** nutritional risk index; **PNI:** prognostic nutritional index; **HALP:** hemoglobin–albumin–lymphocyte and platelet; **ALI:** advanced lung cancer inflammation index; **GINI:** global immune–nutrition-information index; **SIRI:** systemic inflammation response index; **PIIV:** pan-immune–inflammation value. Bold *p* values are statistically significant.

**Table 4:** ROC analysis evaluating the diagnostic performance of scores based on univariate logistic regression in predicting pCR.

Variable	AUC (%95 CI)	*p*	Cutoff	Sensitivity (%)	Specificity (%)
**NRI**	0.840 (0.784–0.895)	**<0.001**	≥126.1	85.34%	83.33%
**(B) PNI**	0.577 (0.504–0.650)	**0.041**	≥59.0	39.66%	75.00%
**(B) HALP**	0.591 (0.519–0.664)	**0.015**	≥48.6	54.31%	64.17%
**(B) ALI**	0.677 (0.609–0.745)	**<0.001**	≥64.8	72.41%	58.33%
**ΔSIRI**	0.617 (0.504–0.950)	**0.011**	≥0.123	60.66%	56.81%
**ΔPIIV**	0.611 (0.532–0.754)	**0.012**	≥−5.70	60.31%	59.38%
**ΔGINI**	0.807 (0.609–0.945)	**<0.001**	≥2.21	80.41%	80.13%

**AUC:** area under the curve; **CI:** confidence interval; **pCR:** pathological complete response; **(B):** before neoadjuvant chemotherapy; **Δ:** (A) − (B); **NRI:** nutritional risk index; **PNI:** prognostic nutritional index; **HALP:** hemoglobin–albumin–lymphocyte and platelet; **ALI:** advanced lung cancer inflammation index; **SIRI:** systemic inflammation response index; **PIIV:** pan-immune–inflammation value; **GINI:** global immune–nutrition-information index. Bold *p* values are statistically significant.

## Data Availability

The datasets used and/or analyzed during the current study are available from the corresponding author on reasonable request.

## Abbreviations

The following abbreviations are used in this manuscript:

pCR	Pathological complete response
NACT	Neoadjuvant chemotherapy
NLR	Neutrophil-to-lymphocyte ratio
BMI	Body mass index
ALI	Advanced lung cancer inflammation index
SII	Systemic immune–inflammation index
PNI	Prognostic nutritional index
PLR	Platelet-to-lymphocyte ratio
SIRI	Systemic inflammation response index
PIIV	Pan-immune–inflammation value
HALP	Hemoglobin–albumin–lymphocyte and platelet
NRI	Nutritional risk index
GINI	Global immune–nutrition-information index
